# Recent Advances in Therapeutic Modalities Against Breast Cancer-Related Lymphedema: Future Epigenetic Landscape

**DOI:** 10.1089/lrb.2022.0016

**Published:** 2023-12-22

**Authors:** Kuo Chen, Narasimha M. Beeraka, Xinliang Zhang, Mikhail Y. Sinelnikov, Maria Plotnikova, Cuiping Zhao, Vijaya Basavaraj, Jin Zhang, Pengwei Lu

**Affiliations:** ^1^ Department of Breast Surgery, The First Affiliated Hospital of Zhengzhou University, Zhengzhou, China.; ^2^ Raghavendra Institute of Pharmaceutical Education and Research (RIPER), Anantapuramu, Andhra Pradesh, India.; ^3^ I.M. Sechenov First Moscow State Medical University of the Ministry of Health of the Russian Federation (Sechenov University), Moscow, Russia.; ^4^ The 80th Army Hospital of the Chinese People's Liberation Army, Weifang, China.; ^5^ Department of Pathology, JSS Medical College, JSS Academy of Higher Education & Research (JSS AHER), Mysuru, Karnataka, India.

**Keywords:** epigenetics, breast cancer-related lymphedema, biomarkers, genetics, therapies, surgery, lymphangiogenesis pathology

## Abstract

**Background:**

Lymphedema is a significant postsurgical complication observed in the majority of breast cancer patients. These multifactorial etiopathogenesis have a significant role in the development of novel diagnostic/prognostic biomarkers and the development of novel therapies. This review aims to ascertain the epigenetic alterations that lead to breast cancer-related lymphedema (BCRL), multiple pathobiological events, and the underlying genetic predisposing factors, signaling cascades pertinent to the lapses in effective prognosis/diagnosis, and finally to develop a suitable therapeutic regimen.

**Methods and Results:**

We have performed a literature search in public databases such as PubMed, Medline, Google Scholar, National Library of Medicine and screened several published reports. Search words such as epigenetics to induce BCRL, prognosis/diagnosis, primary lymphedema, secondary lymphedema, genetic predisposing factors for BRCL, conventional therapies, and surgery were used in these databases. This review described several epigenetic-based predisposing factors and the pathophysiological consequences of BCRL, which affect the overall quality of life, and the interplay of these events could foster the progression of lymphedema in breast cancer survivors. Prognosis/diagnostic and therapy lapses for treating BCRL are highly challenging due to genetic and anatomical variations, alteration in the lymphatic vessel contractions, and variable expression of several factors such as vascular endothelial growth factor (VEGF)-E and vascular endothelial growth factor receptor (VEGFR) in breast cancer survivors.

**Conclusion:**

We compared the efficacy of various conventional therapies for treating BCRL as a multidisciplinary approach. Further substantial research is required to decipher underlying signaling epigenetic pathways to develop chromatin-modifying therapies pertinent to the multiple etiopathogenesis to explore the correlation between the disease pathophysiology and novel therapeutic modalities to treat BCRL.

## Introduction

Breast cancer is one of the most life-threatening causes of death in women worldwide and ∼1.67 million new cases are diagnosed every year.^1–6^ Meanwhile, breast cancer-related lymphedema (BCRL) is one of the significant complications that occur in breast cancer patients after the treatment for breast cancer; it has been reported that more than 20% of patients who undergo treatment for breast cancer may develop BCRL.^7,8^ This disease can occur in patients receiving treatment for solid tumors, with a reported incidence of 16% in melanoma, 30% in sarcoma, 20% in gynecologic tumors, 10% in genitourinary tumors, and 4% in head and neck cancers.^9–11^

Mainly, breast cancer patients after radical mastectomy^12–14^ are more prone to the development of lymphedema. The affected region could cause distressful life in the patients, resulting in functional problems, decreased Quality of Life (QoL), and recurrent infections. The significant pathogenesis of this disease involves dysfunctional lymph transport, and as a result, accumulation of immune cells, lipids, and interstitial fluid in the affected region occurs.^15,16^ Lymphedema is categorized into primary lymphedema and secondary lymphedema; primary lymphedema is induced by the malfunction of the lymphatic system, whereas secondary lymphedema is due to the iatrogenic process. Secondary lymphedema is most significantly observed in cancer therapies.^8^

**FIG. 1.**
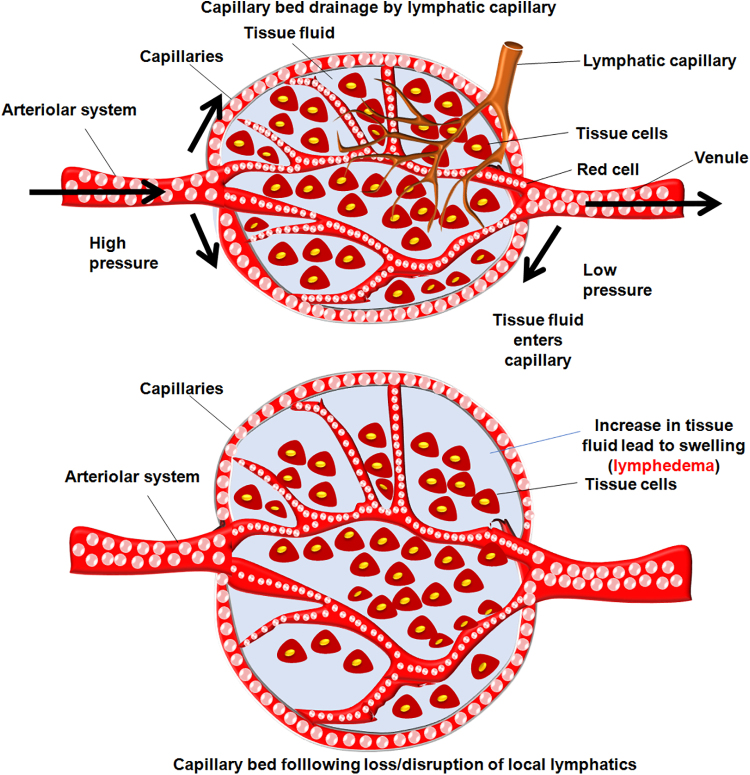
The pathophysiology of lymphedema across arterioles and venules in the lymphatic system of BCRL patients. Damage to the lymphatic vessels through inflammation and adipose tissue deposition in the capillary beds of the lymphatic system in postsurgical BCRL patients could promote the increase in tissue fluid and fosters the formation of fibrosis and swelling. BCRL, breast cancer-related lymphedema.

Furthermore, the lymph system is responsible for draining lymph fluid into the circulation; during lymphedema, the lymph fluid is accumulated in the interstitial space when the lymph drainage is impaired, which consequently causes edema and fosters subcutaneous tissue swelling (Fig. 1).^17^ Lymphedema can induce dysfunction of upper extremities, discomfort, pain, heaviness, psychological changes, unsatisfactory cosmesis, and recurrent infections,^18–21^ eventually exacerbating the poor QoL.^22^ The risk of BCRL is multifactorial, which is due to intake of chemotherapy, radiation therapy, axillary lymph node dissection, advanced disease stage, a substantial number of positive lymph nodes (>8), and a higher BMI (≥25 kg/m^2^).^23,24^

Furthermore, sentinel lymph node biopsy, radiotherapy, obesity, and recurrent infections could cause a clinically relevant lymphedema risk.^25–27^ The incidence of this disease is significantly correlated to the survival time after chemotherapy or radiotherapy.^28^ Therefore, at present, there is a lack of perfect scientific evidence for the pathophysiology and treatment plan for lymphedema. However, BCRL is an incurable condition and current therapies, physical therapies, namely, manual lymph node drainage, shock wave therapy, laser therapy, Qigong-based exercise, and surgical interventions such as derivative microsurgery, vascularized lymph node transfer, microsurgical reconstruction are reported. Yet, due to the lack of sufficient diagnostic strategies, it is challenging to identify presurgical individuals who are at a high risk to develop lymphedema.^7,29–33^

In this review, we critically delineated the need to explore the epigenetic landscape of lymphedema pathophysiology to develop gene-based therapies, discussed the challenges in developing effective prognosis/diagnosis strategies and multiple etiopathogenesis of lymphedema in breast cancer survivors, and also discussed the comparative efficacy of novel therapies (Table 1) for lymphedema treatment.

**Table 1.** Comparison of Different Therapies for Breast Cancer-Related Lymphedema

Methods	Advantages	Disadvantages	Applications	Comments
Complex decongestive therapy	Long-standing experience	Outcome lower-than-expected, poor patient compliance, inconvenience, expensive	MLD, compression therapy, LRE, skin care, and self-management	A current standard protocol for BCRL
Low-level laser therapy	Noninvasive and safe	Outcome lower-than-expected	Phototherapy with wavelengths of light 650–1000 nm	Applied for treating BCRL in the past 20 years
Extracorporeal shock wave therapy	Potential for treating BCRL	Still experimental for BCRL	Stimulation of lymphangiogenesis	Used in articular and ligamental diseases
Hyperbaric oxygen therapy	Potential for treating BCRL	Still experimental for BCRL	100% oxygen inspiration	Applied in several clinical scenarios
Stellate ganglion block	Potential for treating BCRL	Still experimental for BCRL	Local anesthetics	Applied in several clinical scenarios
Pneumatic compression device	As an adjuvant management for BCRL	Outcome lower-than-expected	Sequential compression device	Applied in several clinical scenarios
Mesenchymal stem cell therapy	Potential for treating BCRL	Still experimental for BCRL	Developing angiogenesis and neovascularization	Applied in several clinical scenarios
Surgical management	Effective	Invasive and risk of complications	Lymph reconstruction	Applied from early stage to advanced stage of BCRL
Acupuncture	Potential for treating BCRL	Still experimental for BCRL	Chinese Traditional Medicine	Can be used as an adjunct therapy
Kinesio tape	Potential for treating BCRL	Still experimental for BCRL	An elastic strip with acrylic adhesive	Used for relieving pain and disability from injuries

BCRL, breast cancer-related lymphedema; LRE, lymph-reducing exercises; MLD, manual lymph drainage.

## Literature Search

The literature search was performed extensively to extract the published reports using key words such as epigenetics, gene markers, breast cancer, primary lymphedema, secondary lymphedema, genetic predisposing factors for BRCL, biomarkers, conventional therapies, and surgery, the pathophysiology of BCRL, therapies, diagnostic reports in public databases such as Medline, PubMed, National Library of Medicine, and Good Scholar, which were peer reviewed.

## Biomarkers Based on BCRL Histopathology

The lack of effective prognostic/early diagnostic strategies for BCRL in lymphedema patients could facilitate the advanced stages of lymphedema, in which classical methods of symptomatic therapy do not lead to a viable result. Moreover, to date, there is no full understanding of the cause of specific tissue changes associated with lymphedema.^34^ It is known that the impaired lymph outflow leads to disorganization of the lymphatic bed from the distal to the lesion site. However, the morphological changes (progressive lymphangiosclerosis) are substantially higher in BCRL conditions due to the anatomically justified consequences of impaired lymphatic outflow.^25^

Thus, progressive changes in lymphatic vessels could occur in the collector zone with minimal trauma to the central lymphatic collectors (regional lymph nodes). This process is induced by the uncontrolled division of lymphatic endothelial cells (LECs) in the early stages of lymphedema. However, the markers^35^ and histopathological changes during the early stages have not been examined yet through clinical studies.

During surgical dissection of lymph nodes, in the distal bed, the lymphatic capillaries experience a progressive disturbance to the outflow and confer to the formation of retrograde lymph movement, and infarction of regional collectors, which further reduce the amplitude of vessel contraction at a constant frequency, eventually leading to disorganization of the lymphatic bed as a whole. Ly6G^+^ and CD4^+^ immunocytes are actively involved in the progression of such pathological changes.^36^ Therapeutic strategies to mitigate these pathological changes are limited. Personalization of treatment is a natural stage in the evolution of novel treatment methods against BCRL-induced chronic progressive pathologies.

To develop methods for the personalizing treatment for lymphedema, it is necessary to study the prognosis/diagnosis based on the genetic and proteomic expression pertaining to inflammation and the mitochondrial redox system as these are considered significant prognostic factors that provoke and maintain tissue disorganization and restructuring. Individually variable markers can directly affect the severity of a given disease.^37^ To develop a personalized and high-technology approach to the treatment of lymphedematous pathology, it is necessary to systematize the molecular-genetic and morphological factors affecting the progression of lymphedema and the maintenance of specific pathological changes in the affected tissues.^37^

The etiopathogenesis of lymphedema is not fully understood, however, there is an assumption that individual genetic, immune, and morphological features play a direct role in the pathogenesis of lymphedema and in the abundance of variations in the severity of the disease.^37^ According to previous research reports, up to 80% of patients who underwent radical antitumor treatment develop lymphedema after several months. Furthermore, lymphedema may be developed from 10 days to 30 years after breast cancer treatment. Based on this, we can conclude that the development of lymphedema is based on secondary damage to the lymphatic outflow pathways, which leads to several pathological processes with the development of peripheral lymphadenopathy, and the disruption of metabolic processes in the interstitial space.

Current therapeutic modalities are very limited to treat secondary lymphedema, and therefore, it can cause extensive financial burden and psychological stress in BCRL patients. Therefore, novel treatment strategies are yet to be developed to enhance the overall QoL in these patients.^38^ The efficacy of human hormone components, namely, adrenomedullin, intermedin, and their cognate receptors such as CLR/RAMP1–3 modulate lymphangiogenesis, enhance the LEC permeability and the activity of adipose-tissue derived progenitor cells (ADPCs), and consequently promote lymphatic vessel formation in lymphedema models.^38^ This report hypothesized that the combinatorial regimen of CLR/RAMP receptor ligands with ADPCs could be considered a significant therapeutic strategy to mitigate the devastating pathophysiology of lymphedema. Substantial studies using a combinatorial regimen should be examined for the lymph vessel regenerative capacity using *in vivo* mouse models of secondary lymphedema.^38^

## Lymphedema Pathophysiology and Recent Diagnostic Reports

Dermal thickening is more significantly apparent in the later stages of this disease and confers the formation of hyperkeratosis, acanthosis, lichenification, and verrucae.^39^ Furthermore, cellulitis, lymphangitis, and erysipelas are typically associated with chronic lymphedema.^39^ As per the International Society of Lymphology, stage 0 delineates subclinical lymphedema accompanied by swelling and heaviness in the limb whereas stage I indicates limb swelling and pitting without dermal fibrosis and stage II is characterized by dermal fibrosis. Stage III lymphedema to the most severe stage is accompanied by limb swelling, fat deposits, acanthosis, and verrucae.^39^ A plethora of studies described the involvement of proinflammatory and inflammatory pathways in triggering the pathogenesis of lymphedema. For instance, CD4^+^ T cells such as Th1 and Th2 immune paradigms can trigger the release of IL-4, IL-13, and TGF-β1 across tissues and they can control collagen deposition and fibrosis in lymphedema.^40^

*In vivo* mouse models and BCRL clinical models have already depicted this paradigm pertaining to the pathogenesis of lymphedema. When these mouse models were treated with neutralizing antibodies to neutralize IL-4 or IL-13, lymphedema was reported to be prevented.^41^ Mainly, TGF-β1 has significant implications as the diagnostic marker in lymphedema as it could foster fibrosis, and the administration of TGF-β1 neutralizing antibodies has mitigated fibrosis in *in vivo* models and also impaired the migration of Th2 cells and expression of Th2 cytokines.^42^ However, these mechanisms still require additional research to illustrate the Th2-Th1 immune paradigm in the progression of lymphedema in human models. Furthermore, the accumulation of lymph during lymphedema is associated with the progression of adipogenesis and miscellaneous inflammatory cascades, which could result in complete failure of lymphatic impairment due to the blockade of collateral lymphatic formation.

## Genetics and Lymphangiogenesis in BCRL

**FIG. 2.**
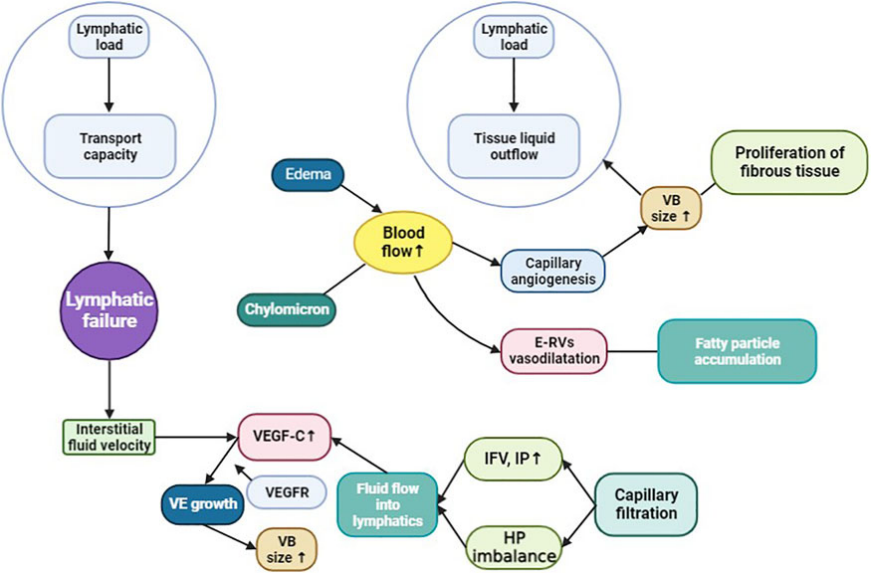
Pathophysiology of lymphedema: Lymphatic load into the lymphatic tissue is enhanced during the postsurgical period in breast cancer patients; and this flow is determined by both intrinsic and extrinsic forces, which fosters the lymph propulsion in the lymphatic conduct; a sequential enhancement in VEGFC and VEGFR activity,^175^ which could confer microvascular permeability across VB and capillary angiogenesis, and promote the overload of the remaining lymphatic drainage capacity. Chylomicrons are the lipid-rich triglyceride-loaded content that enters into the blood circulation via the lymphatic system and the chylomicrons enter into lymphatics, which confers higher lymphedema during BRCL conditions. These events could enhance the proliferation of fibrous tissue in the lymphedema tissue.^23^ E-RVs, existing resistance vessels; HP imbalance, the imbalance of hydrostatic pressure difference; IFV, interstitial fluid volume; IP, interstitial pressure; VB, vascular bed; VE, vascular endothelial; VEGFC, vascular growth endothelial factor C; VEGFR, vascular endothelial growth factor receptor.

Gene-related alterations can promote defects in lymphangiogenesis, and lymphatic function and these mechanisms^29^ yet require substantial research studies in BCRL patients. For instance, the LCP2 is reported to be involved in immune responses by modulating the T cell-induced molecular signaling,^43^ and this gene has a prominent role in the development of lymphatic structures and promotes platelet-dependent mechanism pertinent to the embryogenesis accompanying the separation of blood and lymphatic vessels.^44,45^ Copy number alterations in LCP2 and the tendency of these alterations can significantly induce their influence on other genes known to be implicated in BCRL such as interleukins, IL-10, IL-4, IL-13, and neuropilin 2.^46,47^ NRP2 expression can be observed in LECs and it is upregulated in BCRL patients and acts as a significant mediator to promote angiogenesis and lymphangiogenesis through VEGFC (Fig. 2).

NRP2 is highly expressed during ischemia/hypoxia conditions.^48–50^ Somatic alterations in NRP2, Fms-related tyrosine kinase 4, and VEGFC are typically observed in breast cancer patients^51,52^ and these alterations exhibit a strong tendency to facilitate secondary lymphedema.^53–55^

## Traditional Therapies for Lymphedema-Complex Decongestive Therapy

Complex decongestive therapy (CDT) is also known as combined physical therapy; this therapy is a current standard protocol for treating BCRL, which consists of manual lymph drainage, compression therapy (consisting of compression bandages spiral-bandaging method, and a figure-of-eight method,^56^ compression sleeves, or other types of compression garments), lymph-reducing exercises, skin care, and education on self-management of lymphedema.^7,23,57,58^ The experience of CDT is long-standing.^59^ For instance, there are two phases of CDT (Tables 1 and 2) during treatment; among them, phase 1 of CDT is for reducing swelling and phase 2 is for optimizing the effectiveness of CDT. CDT can significantly reduce the accumulated lymph volume in BCRL conditions, which accompany the mitigation in intensity of pain, heaviness of upper extremities, the incidence of cellulitis, and regaining function of lymph nodes, subsequently improving the overall QoL.^7,60–62^

**Table 2.** Phases of Complex Decongestive Therapy^59^

Phase	Detailed information
“Intensive” phase	Consists of skin care, specific MLD, range of motion exercises, and compression with multilayer compression bandaging.
“Maintenance” phase	Consists of wearing compression garments, regular exercises with the garment worn, and proper skin care. Began promptly after phase 1, with the aim of conserving and optimizing the results.

However, the clinical outcome with CDT is always lower-than-expected in clinical practice; in addition, patient compliance is poor in CDT for BCRL^63^ due to laborious, time-consuming, and expensive processes.^64^ Furthermore, being overweight can mitigate the efficiency of CDT^65^ because the excess adipose tissue may promote the development of chronic massive lymphedema, which cannot be treated by CDT; this therapy cannot eliminate adipose tissue-induced pathophysiology in BCRL conditions by compression alone.^66^ In addition, regular lymphedema treatment can cause an immense financial burden on patients, and the costs are more than $14,000 a year per patient.^67^ There are several surgeons around the world recommending complementary and alternative therapies for treating BCRL.

## Complementary and Alternative Therapies

### Low-level laser therapy

Low-level laser therapy (LLLT) or photobiomodulation has been applied for treating BCRL over the past 20 years. Compared with CDT or surgical management, LLLT is not invasive.^68^ This strategy can be used as a phototherapy using wavelengths of light in the range of 650 to 1000 nm. When the light of LLLT is delivered to the site of BCRL, it can inhibit the inflammation and fibrosis, and consequently decompose the scar, restore the mobility of upper extremities, and regenerate lymph-vessel BCRL conditions.^69–74^ However, a recent study demonstrated that the combinatorial regimen of LLLT combined with conventional CDT has failed to show additional therapeutic benefits.^75^

#### Extracorporeal shock wave therapy

Extracorporeal shock wave therapy (ESWT) could be used in articular and ligamental diseases, such as plantar fasciitis, lateral epicondylitis, and tendinitis.^76,77^ The exact mechanism of ESWT remains unknown. Based on the results of previous animal experiments, ESWT can induce upregulation of angiogenesis-related growth factors including endothelial nitric oxide synthase, nitric oxide, vascular growth endothelial factor (VEGF), bone morphogenetic protein, basic fibroblast growth factor (b-FGF), and proliferating cell nuclear antigen, sequentially promoting neovascularization. These events can enhance blood supply and cell proliferation, and eventually foster tissue regeneration to repair the tendon and articular or bone tissues. ESWT can also be recommended in treating skin ulcers, wounds, scars, necrosis, myocardial ischemia, and muscle spasticity induced through neurological lesions and parodontopathies.^78–80^

The expressions of VEGFC (rat-tail and rabbit-ear models), b-FGF (rat-tail model), and VEGF receptor 3 (VEGFR3, rabbit-ear model) could be induced by low-energy ESWT and stimulate lymphangiogenesis to improve lymphedema in experimental models of rat tails and rabbit ears.^76,80^ A recent pilot study reported that ESWT could reduce the lymph volume in lymphedema conditions and improve the overall QoL in most of the patients with BCRL.^77^ Another study delineated the reduction of lymphedema on long-term ESWT for 6 months.^64^ These studies demonstrated the potential role of ESWT in the treatment of BCRL.

### Hyperbaric oxygen therapy

The hyperbaric oxygen treatment can contribute to the enhanced barometric pressure in the patient's entire body and promote the patient with 100% oxygen for a certain period.^81^ The exact mechanism of hyperbaric oxygen therapy (HBOT) is still unknown, but a pilot study pointed out that the VEGF activity may be stimulated by HBOT.^82^ In another study, fibrosis formation could be inhibited by HBOT and reported to benefit overall improvement in lymphedema conditions.^83^ Furthermore, HBOT can be applied in a lot of clinical scenarios such as carbon monoxide intoxication, decompression sickness, gas gangrene, necrosis, ulcers, hemorrhagic cystitis, proctitis, multiple sclerosis, and vascular dementia. HBOT can also be applied to treat radiation-induced inflammation, such as radiation enteritis, colitis, myelitis, rectosigmoiditis, and brachial plexopathy.^84–102^ Patients' overall QoL is significantly higher with HBOT for mitigating radiation-induced lesions in other organs; meanwhile, this therapy can also reduce lymphedema formation after radiotherapy for breast cancer.^83,103^

However, randomized trials have failed to confirm the positive effect of HBOT in patients with lymphedema.^103^ The effectiveness of HBOT alone for BCRL still needs to be confirmed; the combination therapy of CDT and HBOT, unlike LLLT, is more beneficial than CDT only; therefore, HBOT may be considered an adjunct therapy with conventional treatments such as CDT for patients with BCRL.^104^

### Stellate ganglion block

A stellate ganglion block (SGB) is another therapeutic modality in which the injection of local anesthetic drugs could be used to block the sympathetic nerves located around the cervical sympathetic trunk.^105^ SGB has been used to treat various diseases and disease-induced complications, such as complex regional pain syndrome, hot flashes after breast cancer treatment, and posttraumatic stress disorder; SGB therapy could be used for healing of the fractures, immunologically linked disorders, and for promoting the postoperative recovery of gastrointestinal function; other disease-induced complications such as postoperative trigeminal neuropathy, ventricular arrhythmias, drug-refractory electrical storm, vascular calcification, neuralgia, and vascular insufficiency syndromes of the upper extremity can also be mitigated using this SGB therapeutic modality.^106–120^

Therefore, SGB therapy can be used as an alternative treatment for BCRL.^105^ Furthermore, corticosteroid administration may have an additional benefit in SGB therapy for treating BCRL.^121^ However, substantial research is yet to be performed to explore the underlying pharmacological mechanisms of SGB therapy for treating BCRL.

### Pneumatic compression device

A pneumatic compression device (PCD) is used as an adjuvant therapy to mitigate lymphedema to improve the overall quality of life in BCRL patients.^66^ Therapy involving PCD can reduce lymph volume during lymphedema. So, the frequency of visits, treatments, and complications after discharge of breast cancer survivors can be minimized with regular PCD therapy, and this method is a cost-effective intervention.^122^ However, a meta-analysis reported that a combinatorial regimen of CDT and PCD failed to produce additive efficacy for mitigating lymphedema-induced pathophysiology.^123^

### Mesenchymal stem cell therapy

LECs can undergo differentiation induced from stem cells to promote angiogenesis and neovasculature, consequently conferring the generation of new lymphatic vessels, recovery of impaired lymphatic networks, and reconstruction of the lymphatic circulation pathway.^124^ Adipose-derived stromal stem cells (ASCs) also play a significant role in the treatment of lymphedema. Some reports delineated that the administration of ASCs are beneficial for enhancing the clinical outcomes in BCRL patients due to the reduction in the overall volume of the lower extremity and minimal requirement for subsequent compression therapy. There are no serious adverse effects observed in these patients.^125,126^ ASCs can stimulate the regeneration of lymphatic vessels and enable restoration of the disrupted lymphatic circulation in a murine tail lymphedema model^127^ as ASCs exhibit the pluripotent capacity to differentiate into multiple cell lineages; therefore, the differentiation of ASCs can be conducive to the formation of LECs across deteriorated lymph tissue.

Expression of VEGF-E and VEGFR3 is significantly higher during the process of ASC differentiation to LECs, which further enhances lymphangiogenesis.^128,129^ Thus, stem cell therapy is a promising strategy for the treatment of BCRL, however, clinical trials are required to decipher and confirm the efficacy of this stem cell therapy in the management of BRCL.^124^

### Surgical management

Surgical management of BRCL can be done by lymphovenous anastomosis (earlier stage BCRL) and vascularized lymph node transfer (advanced stage BCRL) to reconstruct the lymph network.^130–132^ A few studies reported that breast reconstruction by deep inferior epigastric perforator-flap or greater omentum flap with vascularized lymph node transfer from inguinal or mesenteric lymph nodes is promising.^131,133,134^ However, surgical intervention is always invasive and it can cause secondary adverse effects to the patients who suffered breast cancer resection. The complications including postoperative infection also cannot be overlooked.

### Acupuncture

Acupuncture as a therapeutic modality has been accepted by modern clinical medicine and used to treat a variety of diseases, such as chronic pain management, osteoarthritis, migraine, anxiety, hot flashes in postmenopausal women, chronic fatigue, neuropathy, nausea, vomiting, xerostomia, and dysphagia.^135–151^ Several clinical trials have proven that acupuncture can enhance a patient's overall QoL by mitigating extremity swelling and lymphedema symptoms.^152–154^ A study at the Memorial Sloan-Kettering Cancer Center (MSKCC) has reported that acupuncture therapy as an adjunct therapy can decrease the overall volume of extremities during lymphedema and minimize the infection rate or other severe side effects.^155^ The efficacy of acupuncture alone still needs to be evaluated for treating BCRL.

### Kinesio-tape

Kinesio tape (K-tape) is an elastic strip with an acrylic adhesive that is used for relieving pain and disability during injuries. The mechanism of K-tape facilitates adhering and lifting the skin in BRCL patients and increases the space beneath the skin and between muscles; therefore, the lymph fluid, interstitial fluid, or blood can flow efficiently and mitigate edema or congestion induced by lymphedema. A randomized clinical trial has shown that K-tape can be a better alternative tool to replace bandages in CDT, especially for patients with poor short-stretch bandage compliance.^156^

## Genetic Variations and Future Epigenetic Alterations in BCRL

Previous evidence described the genetic screening of hereditary syndromes accompanied by the genetic mutations in genes (Table 3) such as FOXC2, FLT-4, and SOX18 for lymphedema-distichiasis, Milroy disease, and hypotrichosis-lymphedema-telangiectasia, respectively. Furthermore, gene mutations such as CCBE1, FAT4, GJC2, VEGFC, PTPN14, GATA2, HGF, and PIEZO1 could induce generalized lymphatic dysplasia, inherited lymphedema type 1C and 1D, lymphedema-choanal atresia, Emberger, and lymphedema-lymphangiectasia, and hereditary lymphedema III, respectively. Hence, future studies require the epigenetic alterations pertinent to these genes triggering the above lymphedema types. In addition, several genetic algorithms were developed to develop genetic markers as molecular diagnostic markers pertinent to BRCL and it is crucial to explore the epigenetic landscape and other chromosomal abnormalities underlying the lymphangio dysplastic syndromes and lymphedema incidence postsurgery in breast cancer conditions.

**Table 3.** Gene Mutations Pertinent to the Clinical Syndromes Where Lymphedema Is a Significant Pathophysiological Component

S. No.	Gene mutations	Clinical syndromes in which “lymphedema” is a significant component	References
1	*PTPN11, KRAS, SOS1*	Noonan syndrome	^174^
2	*MCLMR, KIF11*	Microcephaly–chorioretinopathy–lymphedema-mental retardation	^157^
3	*AKT1*	Proteus syndrome	^157^
4	*PIK3CA*	Fibroadipose hyperplasia	^157^
5	*RASA1*	Park-Weber syndrome (capillary malformation-arteriovenous malformation)	^157^
6	*LRHG, EPHB4*	Lymphatic-related hydrops fetalis	^157^
7	*FOXC2*	Lymphedema-distichiasis	^157^
8	*FLT-4*	Milroy disease	^157^
9	*SOX18*	Hypotrichosis lymphedema-telangiectasia	^157^
10	*CCBE1, FAT4*	Generalized lymphatic dysplasia	^157^
11	*GJC2*	Inherited lymphedema types 1C	^157^
12	*VEGFC*	Inherited lymphedema types 1D	^157^
13	*PTPN14*	Lymphedema-choanal atresia	^157^
14	*GATA2*	Emberger	^157^
15	*GJA1*	Oculodentodigital syndrome	^157^
16	*HGF1*	Lymphedema-lymphangiectasia	^157^
17	*PIEZO1*	Hereditary lymphedema III	^157^

The above clinical syndromes have been associated with lymphedema as a crucial pathophysiological component. Hence, it is highly significant to explore the *de novo* germinal variations pertinent to these genes. Substantial ongoing research using genome-wide association analysis, and whole-Exmore sequencing should focus on the underlying epigenetic landscape of the incidence of secondary lymphedema after breast cancer surgery or mastectomy due to the damage to the lymphatic syndrome. Therefore, it is possible to identify the single/multiple and interacting genetic or epigenetic variants pertinent to the lymphedema incidence for efficient early diagnosis in breast cancer patients.^157^

 Head and neck cancer (HNC) survivors exhibit divergent patterns of methylation in inflammatory signaling, chemokine signaling, TLR-signaling, or natural killer-mediated signaling, and these pathways are also reported to play a significant role in the pathophysiology of postsurgical intervention in HNC patients.^158^ Hence, it is crucial to examine the differentially methylated probes in genes related to these signaling repertoires involved in the lymphedema pathophysiology.

Genetic variation has a significant influence on the incidence of lymphedema in females after mastectomy. The genes such as “GJC2, HGF, and MET” and “IL1A, IL4, IL6, IL10, IL13, VEGFC, NFKB2, LCP-2, NRP-2, SYK, VCAM1, FOXC2, KDR, FLT4, and RORC” were mainly involved in the progression of lymphedema.^159–163^ For instance, mutations occurring in the GJC2 could induce alterations in amino acids in connexin 47, which is associated with lymphedema.^164^ Several single nucleotide polymorphisms are reported to be confined to the coding regions of genes pertinent to lymphedema development and progression.^165,166^

Epigenetic alterations are resulted by alterations in DNA methylation and posttranslational histone alterations. In addition, the changes in noncoding RNA expression could cause epigenetic changes. These alterations are reported to be highly evident in early breast cancer pathogenesis and useful to predict these as biomarkers to foster early detection of breast cancer.^167^ There are no specific reports exploring the specific epigenetic modifications that lead to the pathophysiology of BRCL. DNA methylation factors such as attachment of methyl groups to the CpG nucleotides subsequently form 5-methyl-cytosine in the presence of DNA methyltransferases.^168^ These DNA methylation-mediated epigenetic modifications are yet to be explored in the BCRL pathophysiology. Furthermore, the histone modifications are mainly evident in the H2A, H2B, H3, and H4 proteins.^169^

Posttranslational modifications of histone proteins do not exert any influence on the DNA sequence but influence the transcription process by inducing alterations in the chromatin structure. This can occur by modification of the noncondensed transcriptionally active site to the condensed inactive site. Posttranslational modifications such as sumoylation, DNA methylation, and acetylation are evident in the tail region of these histone proteins.^170^ These alterations are associated with the formation of H3K4me2 and H3K4me3 across the gene region of the promoter, subsequently causing alterations in oncoprotein expression to foster cancers.^171,172^ These histone alterations are yet to be explored in the pathophysiology as these modifications may play a prominent role in the DNA-mediated cellular processes in BCRL.^173^

## Conclusion

Very limited reports are available to delineate the complete pathophysiology and multiple etiopathogenesis of BRCL in breast cancer survivors; therefore, a significant understating of the factors pertinent to the lymphatic system damage in BRCL and the chain of complex progressive events occurring in this condition may benefit the patients by prescribing a suitable combinatorial traditional therapeutic modality. For instance, the accumulated hypertrophic fat moieties can confer the dissipation of lymphatic capillaries consequently inducing damage to the fluid and liquid transport and promoting fat deposition in the periphery of BCRL patients. A better understanding of various gene expression levels is crucial to delineate the single, multiple variants based on the epigenetic landscape of lymphedema; protein-related marker expression is also ascertained to diagnose the disease at an early stage. Furthermore, lymphatic anatomy is crucial to develop novel therapeutic modalities and devices for treating lymphedema.

Yet, large-scale, randomized clinical trials are required to examine their efficacy; in the future, it is crucial to explore the comparative efficacy of the above therapies mentioned in this review to enhance the overall QoL of patients with BCRL.

## References

[1] Chen K, Lu P, Beeraka NM, et al. Mitochondrial mutations and mitoepigenetics: Focus on regulation of oxidative stress-induced responses in breast cancers. Semin Cancer Biol 2020;83:556–569. 33035656 10.1016/j.semcancer.2020.09.012

[2] Ferlay J, Soerjomataram I, Dikshit R, et al. Cancer incidence and mortality worldwide: Sources, methods and major patterns in GLOBOCAN 2012. Int J Cancer 2015;136(5):E359–E386. 25220842 10.1002/ijc.29210

[3] Beeraka NM, Bovilla VR, Doreswamy SH, et al. The taming of nuclear factor erythroid-2-related factor-2 (Nrf2) deglycation by fructosamine-3-kinase (FN3K)-inhibitors—A novel strategy to combat cancers. Cancers (Basel) 2021;13(2):281. 33466626 10.3390/cancers13020281 PMC7828646

[4] Reddy BD, Beeraka NM, Chitturi C, et al. An overview of targeting legumain for inhibiting cancers. Curr Pharm Des 2021;27(31):3337–3348. 33238867 10.2174/1381612826666201125111625

[5] Beeraka NM, Doreswamy SH, Sadhu SP, et al. the role of exosomes in stemness and neurodegenerative diseases—Chemoresistant-cancer therapeutics and phytochemicals. Int J Mol Sci 2020;21(18):6818. 32957534 10.3390/ijms21186818 PMC7555629

[6] Manogaran P, Beeraka NM, Padma VV. The cytoprotective and anti-cancer potential of bisbenzylisoquinoline alkaloids from Nelumbo nucifera. Curr Top Med Chem 2019;19(32):2940–2957. 31738137 10.2174/1568026619666191116160908

[7] Ezzo J, Manheimer E, McNeely ML, et al. Manual lymphatic drainage for lymphedema following breast cancer treatment. Cochrane Database Syst Rev 2015;2015(5):CD003475. 10.1002/14651858.CD003475.pub2 PMC4966288 25994425

[8] Manrique OJ, Bustos SS, Ciudad P, et al. Overview of lymphedema for physicians and other clinicians: A review of fundamental concepts. Mayo Clin Proc 2020;97(10):1920–1935. 32829905 10.1016/j.mayocp.2020.01.006

[9] Lin S, Kim J, Lee M-J, et al. Prospective transcriptomic pathway analysis of human lymphatic vascular insufficiency: Identification and validation of a circulating biomarker panel. PLoS One 2012;7(12):e52021. 23272198 10.1371/journal.pone.0052021 PMC3525657

[10] Manogaran P, Beeraka NM, Huang C-Y, et al. Neferine and isoliensinine enhance ‘intracellular uptake of cisplatin'and induce ‘ROS-mediated apoptosis' in colorectal cancer cells–A comparative study. Food Chem Toxicol 2019;132:110652. 31255669 10.1016/j.fct.2019.110652

[11] Manogaran P, Beeraka NM, Huang C-Y, et al. Neferine and isoliensinine from *Nelumbo nucifera* induced reactive oxygen species (ROS)-mediated apoptosis in colorectal cancer HCT-15 cells. Afr J Pharm Pharmacol 2019;13(8):90–99.

[12] Chen K, Beeraka NM, Gu Y, et al. Totally implantable venous access port systems: Implant depth-based complications in breast cancer therapy—A comparative study. Curr Pharm Des 2021;27(46):4671–4676. 34488584 10.2174/1381612827666210901170522

[13] Chen K, M Beeraka N, Zhang J, et al. Efficacy of da Vinci robot-assisted lymph node surgery than conventional axillary lymph node dissection in breast cancer—A comparative study. Int J Med Robot 2021;17(6):e2307. 34270843 10.1002/rcs.2307

[14] Chen K, Beeraka NM, Li J, Lu P. Anterior abdominal wall defect closed by the anterior sheath of the upper rectus abdominis muscle in a patient with prior “TRAM Flap Breast Reconstruction.” Indian J Surg 2022;84:789–791.

[15] Norman SA, Localio AR, Kallan MJ, et al. Risk factors for lymphedema after breast cancer treatment. Cancer Epidemiol Prev Biomark 2010;19(11):2734–2746. 10.1158/1055-9965.EPI-09-1245 PMC2976830 20978176

[16] McLaughlin SA, Wright MJ, Morris KT, et al. Prevalence of lymphedema in women with breast cancer 5 years after sentinel lymph node biopsy or axillary dissection: Patient perceptions and precautionary behaviors. J Clin Oncol 2008;26(32):5220. 18838708 10.1200/JCO.2008.16.3766 PMC2652092

[17] Morrell RM, Halyard MY, Schild SE, et al. Breast cancer-related lymphedema. Mayo Clin Proc 2005;80(11):1480–1484. 16295027 10.4065/80.11.1480

[18] Brennan MJ, DePompolo RW, Garden FH. Focused review: Postmastectomy lymphedema. Arch Phys Med Rehabil 1996;77(3 Suppl):S74–S80. 8599548 10.1016/s0003-9993(96)90248-8

[19] McWayne J, Heiney SP. Psychologic and social sequelae of secondary lymphedema: A review. Cancer (Basel) 2005;104(3):457–466. 10.1002/cncr.21195 15968692

[20] Smoot B, Wong J, Cooper B, et al. Upper extremity impairments in women with or without lymphedema following breast cancer treatment. J Cancer Surviv 2010;4(2):167–178. 20373044 10.1007/s11764-010-0118-x PMC2882040

[21] Schmitz KH, Ahmed RL, Troxel AB, et al. Weight lifting for women at risk for breast cancer-related lymphedema: A randomized trial. JAMA 2010;304(24):2699–2705. 21148134 10.1001/jama.2010.1837

[22] Pinto M, Gimigliano F, Tatangelo F, et al. Upper limb function and quality of life in breast cancer related lymphedema: a cross-sectional study. Eur J Phys Rehabil Med 2013;49(5):665–673. 23698473

[23] He L, Qu H, Wu Q, et al. Lymphedema in survivors of breast cancer. Oncol Lett 2020;19(3):2085–2096. 32194706 10.3892/ol.2020.11307 PMC7039097

[24] Nguyen TT, Hoskin TL, Habermann EB, et al. Breast cancer-related lymphedema risk is related to multidisciplinary treatment and not surgery alone: Results from a large cohort study. Ann Surg Oncol 2017;24(10):2972–2980. 28766228 10.1245/s10434-017-5960-x PMC5737818

[25] Kwan ML, Darbinian J, Schmitz KH, et al. Risk factors for lymphedema in a prospective breast cancer survivorship study: The Pathways Study. Arch Surg 2010;145(11):1055–1063. 21079093 10.1001/archsurg.2010.231 PMC2997775

[26] Gross JP, Whelan TJ, Parulekar WR, et al. Development and validation of a nomogram to predict lymphedema after axillary surgery and radiation therapy in women with breast cancer from the NCIC CTG MA. 20 randomized trial. Int J Radiat Oncol Biol Phys 2019;105(1):165–173. 31085285 10.1016/j.ijrobp.2019.05.002

[27] Cormier JN, Askew RL, Mungovan KS, et al. Lymphedema beyond breast cancer: A systematic review and meta-analysis of cancer-related secondary lymphedema. Cancer 2010;116(22):5138–5149. 20665892 10.1002/cncr.25458

[28] Li P-J, Jin T, Luo D-H, et al. Effect of prolonged radiotherapy treatment time on survival outcomes after intensity-modulated radiation therapy in nasopharyngeal carcinoma. PLoS One 2015;10(10):e0141332. 26506559 10.1371/journal.pone.0141332 PMC4624640

[29] Invernizzi M, Lopez G, Michelotti A, et al. Integrating biological advances into the clinical Management of Breast Cancer Related Lymphedema. Front Oncol 2020;10:422. 32300557 10.3389/fonc.2020.00422 PMC7142240

[30] Michelotti A, Invernizzi M, Lopez G, et al. Tackling the diversity of breast cancer related lymphedema: perspectives on diagnosis, risk assessment, and clinical management. Breast 2019;44:15–23. 30580170 10.1016/j.breast.2018.12.009

[31] Brorson H. Liposuction in lymphedema treatment. J Reconstr Microsurg 2016;32(1):056–065. 10.1055/s-0035-1549158 25893630

[32] Runowicz CD, Leach CR, Henry NL, et al. American cancer society/American society of clinical oncology breast cancer survivorship care guideline. CA Cancer J Clin 2016;66(1):43–73. 26641959 10.3322/caac.21319

[33] Yamamoto T, Yamamoto N, Hayashi A, et al. Supermicrosurgical deep lymphatic vessel-to-venous anastomosis for a breast cancer-related arm lymphedema with severe sclerosis of superficial lymphatic vessels. Microsurgery 2017;37(2):156–159. 25597913 10.1002/micr.22382

[34] Armer J, Fu M, Wainstock J, et al. Lymphedema following breast cancer treatment, including sentinel lymph node biopsy. Lymphology 2004;37(2):73–91. 15328760

[35] Beylerli O, Beeraka NM, Gareev I, et al. MiRNAs as noninvasive biomarkers and therapeutic agents of pituitary adenomas. Int J Mol Sci 2020;21(19):7287. 33023145 10.3390/ijms21197287 PMC7583927

[36] Todo Y, Yamamoto R, Minobe S, et al. Risk factors for postoperative lower-extremity lymphedema in endometrial cancer survivors who had treatment including lymphadenectomy. Gynecol Oncol 2010;119(1):60–64. 20638109 10.1016/j.ygyno.2010.06.018

[37] Li CY, Kataru RP, Mehrara BJ. Histopathologic features of lymphedema: A molecular review. Int J Mol Sci 2020;21(7):2546. 32268536 10.3390/ijms21072546 PMC7177532

[38] Hsu S-YT. Developing Novel Therapies for Treating Breast-Cancer-Related Lymphedema. National Institute of Health (NIH) Grantome, Adepthera, LLC: Palo Alto, CA; 2022.

[39] Grada AA, Phillips TJ. Lymphedema: Pathophysiology and clinical manifestations. J Am Acad Dermatol 2017;77(6):1009–1020. 29132848 10.1016/j.jaad.2017.03.022

[40] Nores GDG, Ly CL, Cuzzone DA, et al. CD4+ T cells are activated in regional lymph nodes and migrate to skin to initiate lymphedema. Nat Commun 2018;9(1):1–14. 29773802 10.1038/s41467-018-04418-y PMC5958132

[41] Avraham T, Zampell JC, Yan A, et al. Th2 differentiation is necessary for soft tissue fibrosis and lymphatic dysfunction resulting from lymphedema. FASEB J 2013;27(3):1114–1126. 23193171 10.1096/fj.12-222695 PMC3574290

[42] Avraham T, Daluvoy S, Zampell J, et al. Blockade of transforming growth factor-β1 accelerates lymphatic regeneration during wound repair. Am J Pathol 2010;177(6):3202–3214. 21056998 10.2353/ajpath.2010.100594 PMC2993295

[43] Cantrell DA. T-cell antigen receptor signal transduction. Immunology 2002;105(4):369. 11985657 10.1046/j.1365-2567.2002.01391.x PMC1782684

[44] Bertozzi CC, Schmaier AA, Mericko P, et al. Platelets regulate lymphatic vascular development through CLEC-2–SLP-76 signaling. Blood 2010;116(4):661–670. 20363774 10.1182/blood-2010-02-270876 PMC3324297

[45] Bertozzi CC, Hess PR, Kahn ML. Platelets: Covert regulators of lymphatic development. Arterioscler Thromb Vasc Biol 2010;30(12):2368–2371. 21071706 10.1161/ATVBAHA.110.217281 PMC2994722

[46] Abtahian F, Guerriero A, Sebzda E, et al. Regulation of blood and lymphatic vascular separation by signaling proteins SLP-76 and Syk. Science 2003;299(5604):247–251. 12522250 10.1126/science.1079477 PMC2982679

[47] Sebzda E, Hibbard C, Sweeney S, et al. Syk and Slp-76 mutant mice reveal a cell-autonomous hematopoietic cell contribution to vascular development. Dev Cell 2006;11(3):349–361. 16950126 10.1016/j.devcel.2006.07.007

[48] Herzog Y, Kalcheim C, Kahane N, et al. Differential expression of neuropilin-1 and neuropilin-2 in arteries and veins. Mech Dev 2001;109(1):115–119. 11677062 10.1016/s0925-4773(01)00518-4

[49] Yuan L, Moyon D, Pardanaud L, et al. Abnormal lymphatic vessel development in neuropilin 2 mutant mice. Development 2002;129(20):4797–4806. 12361971 10.1242/dev.129.20.4797

[50] Tan G-K, Cooper-White JJ. Interactions of meniscal cells with extracellular matrix molecules: towards the generation of tissue engineered menisci. Cell Adh Migr 2011;5(3):220–226. 21187716 10.4161/cam.5.3.14463 PMC3210205

[51] Joukov V, Pajusola K, Kaipainen A, et al. A novel vascular endothelial growth factor, VEGF-C, is a ligand for the Flt4 (VEGFR-3) and KDR (VEGFR-2) receptor tyrosine kinases. EMBO J 1996;15(2):290–298. 8617204 PMC449944

[52] Favier B, Alam A, Barron P, et al. Neuropilin-2 interacts with VEGFR-2 and VEGFR-3 and promotes human endothelial cell survival and migration. Blood 2006;108(4):1243–1250. 16621967 10.1182/blood-2005-11-4447

[53] Newman B, Lose F, Kedda M-A, et al. Possible genetic predisposition to lymphedema after breast cancer. Lymphat Res Biol 2012;10(1):2–13. 22404826 10.1089/lrb.2011.0024 PMC3311400

[54] Miaskowski C, Dodd M, Paul SM, et al. Lymphatic and angiogenic candidate genes predict the development of secondary lymphedema following breast cancer surgery. PLoS One 2013;8(4):e60164. 23613720 10.1371/journal.pone.0060164 PMC3629060

[55] Fu MR, Conley YP, Axelrod D, et al. Precision assessment of heterogeneity of lymphedema phenotype, genotypes and risk prediction. Breast 2016;29:231–240. 27460425 10.1016/j.breast.2016.06.023 PMC5014618

[56] Johansson K, Albertsson M, Ingvar C, et al. Effects of compression bandaging with or without manual lymph drainage treatment in patients with postoperative arm lymphedema. Lymphology 1999;32(3):103–110. 10494522

[57] Yesil H, Eyigör S, Caramat İ, et al. Effects of complex decongestive therapy on quality of life, depression, neuropathic pain, and fatigue in women with breast cancer-related lymphedema. Turk J Phys Med Rehabil 2017;63(4):329–334. 31453475 10.5606/tftrd.2017.779 PMC6648073

[58] Mayrovitz HN. The standard of care for lymphedema: Current concepts and physiological considerations. Lymphat Res Biol 2009;7(2):101–108. 19522678 10.1089/lrb.2009.0006

[59] Gradalski T, Ochalek K, Kurpiewska J. Complex decongestive lymphatic therapy with or without vodder II manual lymph drainage in more severe chronic postmastectomy upper limb lymphedema: A randomized noninferiority prospective study. J Pain Symptom Manage 2015;50(6):750–757. 26303187 10.1016/j.jpainsymman.2015.06.017

[60] Mobarakeh ZS, Mokhtari-Hesari P, Lotfi-Tokaldany M, et al. Combined decongestive therapy and reduction of pain and heaviness in patients with breast cancer-related lymphedema. Support Care Cancer 2019;27(10):3805–3811. 30729334 10.1007/s00520-019-04681-9

[61] Moffatt CJ, Doherty DC, Franks PJ, et al. Community-based treatment for chronic edema: An effective service model. Lymphat Res Biol 2018;16(1):92–99. 29432067 10.1089/lrb.2017.0021

[62] Ochalek K, Partsch H, Gradalski T, et al. Do compression sleeves reduce the incidence of arm lymphedema and improve quality of life? Two-year results from a prospective randomized trial in breast cancer survivors. Lymphat Res Biol 2019;17(1):70–77. 30339481 10.1089/lrb.2018.0006

[63] Ko DS, Lerner R, Klose G, et al. Effective treatment of lymphedema of the extremities. Arch Surg 1998;133(4):452–458. 9565129 10.1001/archsurg.133.4.452

[64] Cebicci MA, Sutbeyaz ST, Goksu SS, et al. Extracorporeal shock wave therapy for breast cancer-related lymphedema: A pilot study. Arch Phys Med Rehabil 2016;97(9):1520–1525. 26987620 10.1016/j.apmr.2016.02.019

[65] Duyur Cakıt B, Pervane Vural S, Ayhan FF. Complex decongestive therapy in breast cancer-related lymphedema: Does obesity affect the outcome negatively? Lymphat Res Biol 2019;17(1):45–50. 30281384 10.1089/lrb.2017.0086

[66] Rockson SG. Lymphedema after breast cancer treatment. N Engl J Med 2018;379(20):1937–1944. 30428297 10.1056/NEJMcp1803290

[67] Shih YC, Xu Y, Cormier JN, et al. Incidence, treatment costs, and complications of lymphedema after breast cancer among women of working age: A 2-year follow-up study. J Clin Oncol 2009;27(12):2007–2014. 19289624 10.1200/JCO.2008.18.3517

[68] Lima JGME, de Andrade MF, Bergmann A. Low-level laser therapy in secondary lymphedema after breast cancer: Systematic review. Lasers Med Sci 2014;29(3):1289–1295. 23192573 10.1007/s10103-012-1240-y

[69] Baxter GD, Liu L, Petrich S, et al. Low level laser therapy (photobiomodulation therapy) for breast cancer-related lymphedema: A systematic review. BMC Cancer 2017;17(1):833. 29216916 10.1186/s12885-017-3852-x PMC5719569

[70] Nouri K, Jimenez GP, Harrison-Balestra C, et al. 585-nm pulsed dye laser in the treatment of surgical scars starting on the suture removal day. Dermatol Surg 2003;29(1):65–73; discussion 73. 12534515 10.1046/j.1524-4725.2003.29014.x

[71] Assis L, Moretti AI, Abrahão TB, et al. Low-level laser therapy (808 nm) contributes to muscle regeneration and prevents fibrosis in rat tibialis anterior muscle after cryolesion. Lasers Med Sci 2013;28(3):947–955. 22898787 10.1007/s10103-012-1183-3 PMC3521873

[72] Jang DH, Song DH, Chang EJ, et al. Anti-inflammatory and lymphangiogenetic effects of low-level laser therapy on lymphedema in an experimental mouse tail model. Lasers Med Sci 2016;31(2):289–296. 26714983 10.1007/s10103-015-1854-y

[73] Carati CJ, Anderson SN, Gannon BJ, et al. Treatment of postmastectomy lymphedema with low-level laser therapy: A double blind, placebo-controlled trial. Cancer 2003;98(6):1114–1122. 12973834 10.1002/cncr.11641

[74] Kaviani A, Fateh M, Yousefi Nooraie R, et al. Low-level laser therapy in management of postmastectomy lymphedema. Lasers Med Sci 2006;21(2):90–94. 16673054 10.1007/s10103-006-0380-3

[75] Baxter GD, Liu L, Tumilty S, et al. Low level laser therapy for the management of breast cancer-related lymphedema: A randomized controlled feasibility study. Lasers Surg Med 2018;50(9):924–932. 29851090 10.1002/lsm.22947

[76] Kubo M, Li TS, Kamota T, et al. Extracorporeal shock wave therapy ameliorates secondary lymphedema by promoting lymphangiogenesis. J Vasc Surg 2010;52(2):429–434. 20670777 10.1016/j.jvs.2010.03.017

[77] Bae H, Kim HJ. Clinical outcomes of extracorporeal shock wave therapy in patients with secondary lymphedema: A pilot study. Ann Rehabil Med 2013;37(2):229–234. 23705118 10.5535/arm.2013.37.2.229 PMC3660484

[78] Romeo P, Lavanga V, Pagani D, et al. Extracorporeal shock wave therapy in musculoskeletal disorders: A review. Med Princ Pract 2014;23(1):7–13. 24217134 10.1159/000355472 PMC5586835

[79] Wang CJ. An overview of shock wave therapy in musculoskeletal disorders. Chang Gung Med J 2003;26(4):220–232. 12846521

[80] Serizawa F, Ito K, Matsubara M, et al. Extracorporeal shock wave therapy induces therapeutic lymphangiogenesis in a rat model of secondary lymphoedema. Eur J Vasc Endovasc Surg 2011;42(2):254–260. 21454105 10.1016/j.ejvs.2011.02.029

[81] Kirby JP, Snyder J, Schuerer DJE, et al. Essentials of hyperbaric oxygen therapy: 2019 review. Mo Med 2019;116(3):176–179. 31527935 PMC6690283

[82] Teas J, Cunningham JE, Cone L, et al. Can hyperbaric oxygen therapy reduce breast cancer treatment-related lymphedema? A pilot study. J Womens Health (Larchmt) 2004;13(9):1008–1018. 15665658 10.1089/jwh.2004.13.1008

[83] Gothard L, Stanton A, MacLaren J, et al. Non-randomised phase II trial of hyperbaric oxygen therapy in patients with chronic arm lymphoedema and tissue fibrosis after radiotherapy for early breast cancer. Radiother Oncol 2004;70(3):217–224. 15064005 10.1016/S0167-8140(03)00235-4

[84] Marx RE, Johnson RP, Kline SN. Prevention of osteoradionecrosis: A randomized prospective clinical trial of hyperbaric oxygen versus penicillin. J Am Dent Assoc 1985;111(1):49–54. 3897335 10.14219/jada.archive.1985.0074

[85] Bassetto F, Bosco G, Brambullo T, et al. Hyperbaric oxygen therapy in Plastic Surgery practice: Case series and literature overview. G Chir 2019;40(4):257–275. 32011977

[86] Bevers RF, Bakker DJ, Kurth KH. Hyperbaric oxygen treatment for haemorrhagic radiation cystitis. Lancet 1995;346(8978):803–805. 7674746 10.1016/s0140-6736(95)91620-2

[87] Oscarsson N, Müller B, Rosén A, et al. Radiation-induced cystitis treated with hyperbaric oxygen therapy (RICH-ART): A randomised, controlled, phase 2–3 trial. Lancet Oncol 2019;20(11):1602–1614. 31537473 10.1016/S1470-2045(19)30494-2

[88] Nakada T, Yamaguchi T, Sasagawa I, et al. Successful hyperbaric oxygenation for radiation cystitis due to excessive irradiation to uterus cancer. Eur Urol 1992;22(4):294–297. 1490506 10.1159/000474775

[89] Weiss JP, Mattei DM, Neville EC, et al. Primary treatment of radiation-induced hemorrhagic cystitis with hyperbaric oxygen: 10-year experience. J Urol 1994;151(6):1514–1517. 8189559 10.1016/s0022-5347(17)35289-8

[90] Bem J, Bem S, Singh A. Use of hyperbaric oxygen chamber in the management of radiation-related complications of the anorectal region: Report of two cases and review of the literature. Dis Colon Rectum 2000;43(10):1435–1438. 11052522 10.1007/BF02236641

[91] Hamour AA, Denning DW. Hyperbaric oxygen therapy in a woman who declined colostomy. Lancet 1996;348(9021):197. 10.1016/s0140-6736(05)66143-0 8684174

[92] Luk KH, Baker DG, Fellows CF. Hyperbaric oxygen after radiation and its effect on the production of radiation myelitis. Int J Radiat Oncol Biol Phys 1978;4(5–6):457–459. 689946 10.1016/0360-3016(78)90079-2

[93] Barnes MP, Bates D, Cartlidge NE, et al. Hyperbaric oxygen and multiple sclerosis: short-term results of a placebo-controlled, double-blind trial. Lancet 1985;1(8424):297–300. 2857361 10.1016/s0140-6736(85)91079-7

[94] You Q, Li L, Xiong SQ, et al. Meta-analysis on the efficacy and safety of hyperbaric oxygen as adjunctive therapy for vascular dementia. Front Aging Neurosci 2019;11:86. 31057392 10.3389/fnagi.2019.00086 PMC6478752

[95] Kitta T, Shinohara N, Shirato H, et al. The treatment of chronic radiation proctitis with hyperbaric oxygen in patients with prostate cancer. BJU Int 2000;85(3):372–374. 10671898 10.1046/j.1464-410x.2000.00404.x

[96] Moulin C, Li V, Loizzo F, et al. Value of hyperbaric oxygen in the hemostatic treatment of chronic radiation-induced recto-sigmoiditis. Gastroenterol Clin Biol 1993;17(6–7):520–521. 8243953

[97] Aanderud L, Thorsen E, Brattebø G, et al. Hyperbaric oxygen treatment for radiation reactions [in Norwegian]. Tidsskr Nor Laegeforen 2000;120(9):1020–1022. 10833959

[98] Woo TC, Joseph D, Oxer H. Hyperbaric oxygen treatment for radiation proctitis. Int J Radiat Oncol Biol Phys 1997;38(3):619–622. 9231688 10.1016/s0360-3016(97)00017-5

[99] Warren DC, Feehan P, Slade JB, et al. Chronic radiation proctitis treated with hyperbaric oxygen. Undersea Hyperb Med 1997;24(3):181–184. 9308141

[100] Xiao Y, Wang J, Jiang S, et al. Hyperbaric oxygen therapy for vascular dementia. Cochrane Database Syst Rev 2012;7:CD009425. 10.1002/14651858.CD009425.pub2 PMC11668924 22786527

[101] Xu Y, Wang Q, Qu Z, et al. Protective effect of hyperbaric oxygen therapy on cognitive function in patients with vascular dementia. Cell Transplant 2019;28(8):1071–1075. 31134827 10.1177/0963689719853540 PMC6728711

[102] Pritchard J, Anand P, Broome J, et al. Double-blind randomized phase II study of hyperbaric oxygen in patients with radiation-induced brachial plexopathy. Radiother Oncol 2001;58(3):279–286. 11230889 10.1016/s0167-8140(00)00319-4

[103] Gothard L, Haviland J, Bryson P, et al. Randomised phase II trial of hyperbaric oxygen therapy in patients with chronic arm lymphoedema after radiotherapy for cancer. Radiother Oncol 2010;97(1):101–107. 20605648 10.1016/j.radonc.2010.04.026

[104] Koo JH, Song SH, Oh HS, et al. Comparison of the short-term effects of hyperbaric oxygen therapy and complex decongestive therapy on breast cancer-related lymphedema: A pilot study. Medicine (Baltimore) 2020;99(11):e19564. 32176114 10.1097/MD.0000000000019564 PMC7440127

[105] Park MW, Lee SU, Kwon S, et al. Comparison between the effectiveness of complex decongestive therapy and stellate ganglion block in patients with breast cancer-related lymphedema: A randomized controlled study. Pain Phys 2019;22(3):255–263. 31151333

[106] Yucel I, Demiraran Y, Ozturan K, et al. Complex regional pain syndrome type I: efficacy of stellate ganglion blockade. J Orthop Traumatol 2009;10(4):179–183. 19888550 10.1007/s10195-009-0071-5 PMC2784060

[107] Makharita MY, Amr YM, El-Bayoumy Y. Effect of early stellate ganglion blockade for facial pain from acute herpes zoster and incidence of postherpetic neuralgia. Pain Phys 2012;15(6):467–474. 23159962

[108] Haest K, Kumar A, Van Calster B, et al. Stellate ganglion block for the management of hot flashes and sleep disturbances in breast cancer survivors: an uncontrolled experimental study with 24 weeks of follow-up. Ann Oncol 2012;23(6):1449–1454. 22039079 10.1093/annonc/mdr478

[109] Lipov E, Ritchie EC. A review of the use of stellate ganglion block in the treatment of PTSD. Curr Psychiatry Rep 2015;17(8):599. 26073361 10.1007/s11920-015-0599-4

[110] Rae Olmsted KL, Bartoszek M, Mulvaney S, et al. Effect of stellate ganglion block treatment on posttraumatic stress disorder symptoms: A randomized clinical trial. JAMA Psychiatry 2020;77(2):130–138. 31693083 10.1001/jamapsychiatry.2019.3474 PMC6865253

[111] Kizilay H, Cakici H, Kilinc E, et al. Effects of stellate ganglion block on healing of fractures induced in rats. Biomed Res Int 2020;2020:4503463. 32879882 10.1155/2020/4503463 PMC7448117

[112] Lipov E, Gluncic V, Lukić IK, et al. How does stellate ganglion block alleviate immunologically-linked disorders? Med Hypotheses 2020;144:110000. 32758866 10.1016/j.mehy.2020.110000

[113] Wen B, Wang Y, Zhang C, et al. Effect of stellate ganglion block on postoperative recovery of gastrointestinal function in patients undergoing surgery with general anaesthesia: A meta-analysis. BMC Surg 2020;20(1):284. 33198732 10.1186/s12893-020-00943-0 PMC7670678

[114] Ganesh A, Qadri YJ, Boortz-Marx RL, et al. Stellate ganglion blockade: An intervention for the management of ventricular arrhythmias. Curr Hypertens Rep 2020;22(12):100. 33097982 10.1007/s11906-020-01111-8 PMC7646199

[115] Fudim M, Boortz-Marx R, Ganesh A, et al. Stellate ganglion blockade for the treatment of refractory ventricular arrhythmias: A systematic review and meta-analysis. J Cardiovasc Electrophysiol 2017;28(12):1460–1467. 28833780 10.1111/jce.13324

[116] Tian Y, Wittwer ED, Kapa S, et al. Effective use of percutaneous stellate ganglion blockade in patients with electrical storm. Circ Arrhythm Electrophysiol 2019;12(9):e007118. 31514529 10.1161/CIRCEP.118.007118

[117] Guttuso T Jr. Stellate ganglion block for treating hot flashes: A viable treatment option or sham procedure? Maturitas 2013;76(3):221–224. 24021996 10.1016/j.maturitas.2013.08.001

[118] Hao W, Yang R, Yang Y, et al. Stellate ganglion block ameliorates vascular calcification by inhibiting endoplasmic reticulum stress. Life Sci 2018;193:1–8. 29208463 10.1016/j.lfs.2017.12.002

[119] Abbas DN, Reyad RM. Thermal versus super voltage pulsed radiofrequency of stellate ganglion in post-mastectomy neuropathic pain syndrome: A prospective randomized trial. Pain Phys 2018;21(4):351–362. 30045592

[120] Sago T, Takahashi O, Ogawa M, et al. Effects of stellate ganglion block on postoperative trigeminal neuropathy after dental surgery: A propensity score matching analysis. Sci Rep 2020;10(1):13463. 32778742 10.1038/s41598-020-70533-w PMC7417992

[121] Park JH, Min YS, Chun SM, et al. Effects of stellate ganglion block on breast cancer-related lymphedema: Comparison of various injectates. Pain Phys 2015;18(1):93–99. 25675063

[122] Karaca-Mandic P, Hirsch AT, Rockson SG, et al. The cutaneous, net clinical, and health economic benefits of advanced pneumatic compression devices in patients with lymphedema. JAMA Dermatol 2015;151(11):1187–1193. 26444458 10.1001/jamadermatol.2015.1895

[123] Shao Y, Qi K, Zhou QH, et al. Intermittent pneumatic compression pump for breast cancer-related lymphedema: A systematic review and meta-analysis of randomized controlled trials. Oncol Res Treat 2014;37(4):170–174. 24732640 10.1159/000360786

[124] Chen K, Sinelnikov MY, Reshetov IV, et al. Therapeutic potential of mesenchymal stem cells for postmastectomy lymphedema: A literature review. Clin Transl Sci 2021;14(1):54–61. 33460321 10.1111/cts.12864 PMC7877822

[125] Toyserkani NM, Jensen CH, Sheikh SP, et al. Cell-assisted lipotransfer using autologous adipose-derived stromal cells for alleviation of breast cancer-related lymphedema. Stem Cells Transl Med 2016;5(7):857–859. 27151914 10.5966/sctm.2015-0357 PMC4922856

[126] Toyserkani NM, Jensen CH, Andersen DC, et al. Treatment of breast cancer-related lymphedema with adipose-derived regenerative cells and fat grafts: A feasibility and safety study. Stem Cells Transl Med 2017;6(8):1666–1672. 28653440 10.1002/sctm.17-0037 PMC5689749

[127] Ackermann M, Wettstein R, Senaldi C, et al. Impact of platelet rich plasma and adipose stem cells on lymphangiogenesis in a murine tail lymphedema model. Microvasc Res 2015;102:78–85. 26365474 10.1016/j.mvr.2015.09.001

[128] Yoshida S, Hamuy R, Hamada Y, et al. Adipose-derived stem cell transplantation for therapeutic lymphangiogenesis in a mouse secondary lymphedema model. Regen Med 2015;10(5):549–562. 26237700 10.2217/rme.15.24

[129] Hwang JH, Kim IG, Lee JY, et al. Therapeutic lymphangiogenesis using stem cell and VEGF-C hydrogel. Biomaterials 2011;32(19):4415–4423. 21421266 10.1016/j.biomaterials.2011.02.051

[130] Chen K, Sinelnikov MY, Shchedrina MA, et al. Surgical management of postmastectomy lymphedema and review of the literature. Ann Plast Surg 2021;86(3S Suppl 2):S173–S176. 33346539 10.1097/SAP.0000000000002642

[131] Chang EI, Ibrahim A, Liu J, et al. Optimizing quality of life for patients with breast cancer-related lymphedema: A prospective study combining DIEP flap breast reconstruction and lymphedema surgery. Plast Reconstr Surg 2020;145(4):676e–685e. 10.1097/PRS.0000000000006634 32221193

[132] Chang DW, Suami H, Skoracki R. A prospective analysis of 100 consecutive lymphovenous bypass cases for treatment of extremity lymphedema. Plast Reconstr Surg 2013;132(5):1305–1314. 24165613 10.1097/PRS.0b013e3182a4d626

[133] Sinelnikov MY, Chen K, Sukorceva NS, et al. A clinical case of breast reconstruction with greater omentum flap for treatment of upper extremity lymphedema. Plast Reconstr Surg Glob Open 2019;7(9):e2402. 31942381 10.1097/GOX.0000000000002402 PMC6908388

[134] Coriddi M, Wee C, Meyerson J, et al. Vascularized jejunal mesenteric lymph node transfer: A novel surgical treatment for extremity lymphedema. J Am Coll Surg 2017;225(5):650–657. 28818700 10.1016/j.jamcollsurg.2017.08.001

[135] Crew KD, Capodice JL, Greenlee H, et al. Randomized, blinded, sham-controlled trial of acupuncture for the management of aromatase inhibitor-associated joint symptoms in women with early-stage breast cancer. J Clin Oncol 2010;28(7):1154–1160. 20100963 10.1200/JCO.2009.23.4708

[136] Haake M, Müller HH, Schade-Brittinger C, et al. German Acupuncture Trials (GERAC) for chronic low back pain: randomized, multicenter, blinded, parallel-group trial with 3 groups. Arch Intern Med 2007;167(17):1892–1898. 17893311 10.1001/archinte.167.17.1892

[137] Lee MS, Ernst E. Acupuncture for pain: An overview of Cochrane reviews. Chin J Integr Med 2011;17(3):187–189. 21359919 10.1007/s11655-011-0665-7

[138] Lee SH, Lee BC. Use of acupuncture as a treatment method for chronic prostatitis/chronic pelvic pain syndromes. Curr Urol Rep 2011;12(4):288–296. 21472420 10.1007/s11934-011-0186-0

[139] Shiflett SC, Schwartz GE. Meta-analysis of randomised controlled trials (RCTs) involving acupuncture for labour pain shows acupuncture to be more effective than comparison treatments in several significant circumstances. BJOG 2011;118(1):100–101; author reply 101–102. 10.1111/j.1471-0528.2010.02758.x 21143748

[140] Selfe TK, Taylor AG. Acupuncture and osteoarthritis of the knee: A review of randomized, controlled trials. Fam Community Health 2008;31(3):247–254. 18552606 10.1097/01.FCH.0000324482.78577.0f PMC2810544

[141] Linde K, Allais G, Brinkhaus B, et al. Acupuncture for migraine prophylaxis. Cochrane Database Syst Rev 2009;1:CD001218. 10.1002/14651858.CD001218.pub2 PMC3099267 19160193

[142] Wang SM, Punjala M, Weiss D, et al. Acupuncture as an adjunct for sedation during lithotripsy. J Altern Complement Med 2007;13(2):241–246. 17388767 10.1089/acm.2006.6262

[143] Wang SM, Escalera S, Lin EC, et al. Extra-1 acupressure for children undergoing anesthesia. Anesth Analg 2008;107(3):811–816. 18713889 10.1213/ane.0b013e3181804441

[144] Walker EM, Rodriguez AI, Kohn B, et al. Acupuncture versus venlafaxine for the management of vasomotor symptoms in patients with hormone receptor-positive breast cancer: A randomized controlled trial. J Clin Oncol 2010;28(4):634–640. 20038728 10.1200/JCO.2009.23.5150

[145] Kim KH, Kang KW, Kim DI, et al. Effects of acupuncture on hot flashes in perimenopausal and postmenopausal women—A multicenter randomized clinical trial. Menopause 2010;17(2):269–280. 19907348 10.1097/gme.0b013e3181bfac3b

[146] Vickers AJ, Straus DJ, Fearon B, et al. Acupuncture for postchemotherapy fatigue: A phase II study. J Clin Oncol 2004;22(9):1731–1735. 15117996 10.1200/JCO.2004.04.102

[147] Wong R, Sagar S. Acupuncture treatment for chemotherapy-induced peripheral neuropathy—A case series. Acupunct Med 2006;24(2):87–91. 16783284 10.1136/aim.24.2.87

[148] Molassiotis A, Helin AM, Dabbour R, et al. The effects of P6 acupressure in the prophylaxis of chemotherapy-related nausea and vomiting in breast cancer patients. Complement Ther Med 2007;15(1):3–12. 17352966 10.1016/j.ctim.2006.07.005

[149] Gottschling S, Reindl TK, Meyer S, et al. Acupuncture to alleviate chemotherapy-induced nausea and vomiting in pediatric oncology—A randomized multicenter crossover pilot trial. Klin Padiatr 2008;220(6):365–370. 18949672 10.1055/s-0028-1086039

[150] Pfister DG, Cassileth BR, Deng GE, et al. Acupuncture for pain and dysfunction after neck dissection: Results of a randomized controlled trial. J Clin Oncol 2010;28(15):2565–2570. 20406930 10.1200/JCO.2009.26.9860 PMC2881730

[151] Lu W, Posner MR, Wayne P, et al. Acupuncture for dysphagia after chemoradiation therapy in head and neck cancer: A case series report. Integr Cancer Ther 2010;9(3):284–290. 20713374 10.1177/1534735410378856 PMC3014053

[152] Kanakura Y, Niwa K, Kometani K, et al. Effectiveness of acupuncture and moxibustion treatment for lymphedema following intrapelvic lymph node dissection: A preliminary report. Am J Chin Med 2002;30(1):37–43. 12067095 10.1142/S0192415X02000041

[153] Alem M, Gurgel MS. Acupuncture in the rehabilitation of women after breast cancer surgery—A case series. Acupunct Med 2008;26(2):87–93. 18591908

[154] Cassileth BR, Van Zee KJ, Chan Y, et al. A safety and efficacy pilot study of acupuncture for the treatment of chronic lymphoedema. Acupunct Med 2011;29(3):170–172. 21685498 10.1136/aim.2011.004069 PMC3171073

[155] Cassileth BR, Van Zee KJ, Yeung KS, et al. Acupuncture in the treatment of upper-limb lymphedema: results of a pilot study. Cancer 2013;119(13):2455–2461. 23576267 10.1002/cncr.28093 PMC3738927

[156] Tsai HJ, Hung HC, Yang JL, et al. Could Kinesio tape replace the bandage in decongestive lymphatic therapy for breast-cancer-related lymphedema? A pilot study. Support Care Cancer 2009;17(11):1353–1360. 19199105 10.1007/s00520-009-0592-8

[157] Executive Committee of the International Society of Lymphology. The diagnosis and treatment of peripheral lymphedema: 2016 consensus document of the International Society of Lymphology. Lymphology 2016;49(4):170–184. 29908550

[158] Moody L, Crowder SL, Fruge AD, et al. Epigenetic stratification of head and neck cancer survivors reveals differences in lycopene levels, alcohol consumption, and methylation of immune regulatory genes. Clin Epigenetics 2020;12(1):1–14. 10.1186/s13148-020-00930-5 PMC7488769 32917280

[159] Clark B, Sitzia J, Harlow W. Incidence and risk of arm oedema following treatment for breast cancer: A three-year follow-up study. QJM 2005;98(5):343–348. 15820971 10.1093/qjmed/hci053

[160] Armer JM, Stewart BR. A comparison of four diagnostic criteria for lymphedema in a post-breast cancer population. Lymphat Res Biol 2005;3(4):208–217. 16379589 10.1089/lrb.2005.3.208

[161] Bernas M. Assessment and risk reduction in lymphedema. Semin Oncol Nurs 2013;29(1):12–19. 23375062 10.1016/j.soncn.2012.11.003

[162] Armer JM. Research on risk assessment for secondary lymphedema following breast cancer treatment risk factors for lymphedema after breast cancer. Cancer Epidemiology, Biomark Prev 2010;19(11):2715–2717. 10.1158/1055-9965.EPI-10-0962 20978175

[163] Van Onselen C, Cooper BA, Lee K, et al. Identification of distinct subgroups of breast cancer patients based on self-reported changes in sleep disturbance. Support Care Cancer 2012;20:2611–2619. 22290719 10.1007/s00520-012-1381-3

[164] Morcos B, Ahmad FA, Anabtawi I, et al. Development of breast cancer-related lymphedema: is it dependent on the patient, the tumor or the treating physicians? Surg Today 2014;44:100–106. 23377553 10.1007/s00595-013-0494-8

[165] Jensen MR, Simonsen L, Karlsmark T, et al. Microvascular filtration is increased in the forearms of patients with breast cancer–related lymphedema. J Appl Physiol 2013;114(1):19–27. 23123353 10.1152/japplphysiol.01116.2012

[166] Leung G, Baggott C, West C, et al. Cytokine candidate genes predict the development of secondary lymphedema following breast cancer surgery. Lymphat Res Biol 2014;12(1):10–22. 24502445 10.1089/lrb.2013.0024 PMC3961780

[167] Kanwal R, Gupta S. Epigenetic modifications in cancer. Clin Genet 2012;81(4):303–311. 22082348 10.1111/j.1399-0004.2011.01809.x PMC3590802

[168] Veeck J, Esteller M. Breast cancer epigenetics: From DNA methylation to microRNAs. J Mammary Gland Biol Neoplasia 2010;15:5–17. 20101446 10.1007/s10911-010-9165-1 PMC2824126

[169] Audia JE, Campbell RM. Histone modifications and cancer. Cold Spring Harb Perspect Biol 2016;8(4):a019521. 27037415 10.1101/cshperspect.a019521 PMC4817802

[170] Kurdistani S. Histone modifications as markers of cancer prognosis: A cellular view. Br J Cancer 2007;97(1):1–5. 17592497 10.1038/sj.bjc.6603844 PMC2359665

[171] Horn PJ, Peterson CL. Heterochromatin assembly: A new twist on an old model. Chromosome Res 2006;14:83–94. 16506098 10.1007/s10577-005-1018-1

[172] Yan W, Herman JG, Guo M. Epigenome-based personalized medicine in human cancer. Epigenomics 2016;8(1):119–133. 26344672 10.2217/epi.15.84

[173] Buocikova V, Rios-Mondragon I, Pilalis E, et al. Epigenetics in breast cancer therapy—New strategies and future nanomedicine perspectives. Cancers (Basel) 2020;12(12):3622. 33287297 10.3390/cancers12123622 PMC7761669

[174] Sleutjes J, Kleimeier L, Leenders E, et al. Lymphatic abnormalities in noonan syndrome spectrum disorders: A systematic review. Mol Syndromol 2022;13(1):1–11. 35221870 10.1159/000517605 PMC8832235

[175] Dallavalasa S, Beeraka NM, Basavaraju CG, et al. The role of tumor associated macrophages (TAMs) in cancer progression, chemoresistance, angiogenesis and metastasis-current status. Curr Med Chem 2021;28(39):8203–8236. 34303328 10.2174/0929867328666210720143721

